# Online taxi users' optimistic bias: China youths' digital travel and information privacy protection

**DOI:** 10.3389/fpsyg.2022.1049925

**Published:** 2022-11-22

**Authors:** Xiaoyang Meng, Bobo Feng

**Affiliations:** School of Journalism and Communication, Southwest University of Political Science and Law, Chongqing, China

**Keywords:** privacy practice, privacy concern, privacy knowledge, optimistic bias, digital travel

## Abstract

Digital travel platforms not only provided people with convenient travel but also raised a series of problems regarding information privacy protection. In order to analyze privacy protection behavior, this study surveyed 441 subjects aged 18–35 who utilized digital travel platforms based on a structural model of protective motivation theory. The results indicated that a perceived threat, self-efficacy, and response efficacy positively and significantly impacted youths' privacy concerns. Furthermore, privacy concerns were positively related to privacy protection behavior and were an intermediate variable between the relationships among perceived threat, self-efficacy, response efficacy, and privacy protection behavior. This study identified the moderating effect of youths' knowledge of platform privacy settings on the relationship between privacy concerns and protection behavior. In addition, the results confirmed that an optimistic bias did exist among talented youth with high privacy knowledge in terms of a practical level of privacy management. These unique findings represent the exceptional contributions and innovation points of this study.

## Introduction

Digital travel platforms (DTP) have gradually permeated our daily lives in various fields due to information technology's rapid development and evolution. According to a statistical report of the 49^th^ China Internet Development Status, there were over 4.53 hundred million online taxi users in China, which accounted for 43.9% of Chinese Internet users. However, digital travel platforms not only provided them with convenient travel but also raised a series of problems regarding information privacy protection. Due to various incidents of serious illegal collection of people's personal information known to the public, the National Network Information Office officially shut down 25 digital travel platforms on July 4, 2021. The practical levels of this phenomenon illustrated that online taxi users' personal information was collected unreasonably and illegally, which reflected a tremendous threat to privacy loopholes. According to studies of digital travel platforms, youths account for the majority of customers of online taxis, and college students prefer carpooling. The data in DTP include users' ID card numbers, names, ages, and other information for privacy purposes such as audio/video records during the ride, facial IDs, travel routes, call logs, and sensitive sites. Leakage of information privacy would seriously damage an individual's personal safety, property, and dignity. Thus, this study focused on young users' attitudes toward online travel platforms' information privacy concerns and protection. This study aimed to explore privacy protection theories among youths and offer practical guidance regarding information privacy protection.

Few quantitative studies on youths' attitudes toward digital travel platform information privacy protection were identified, but they were very helpful. Protection motivation theory and social cognitive theory have become significant theories investigating the relationship between perceived online threats and online behavior (Milne et al., 2009). Studies argue that the enhancement of privacy concerns leads to an increase in protection behavior and a decrease in online privacy disclosure (Chen and Chen, 2015). From the perspective of privacy protection, youths' personal information safety behavior in social networks was significantly affected by their perceived threat, self-efficacy, and response efficacy (Wang et al., 2018). Certain studies showed that college students' perceived risk of the WeChat application process significantly triggered their privacy concerns in social networking (Shen, 2017), which illustrated their concerns about information privacy. In addition, the increase in online privacy concerns among youths directly affects their privacy protection behavior and disclosure of information privacy (Jia et al., 2021), which indicates that the privacy protection behavior of the youths was affected by multiple predisposing factors and variables. In contrast to the traditional privacy framework structure, a study asserted that privacy knowledge level was an intermediary factor in the relationship between privacy concerns and self-disclosure behavior (Qiang and Xiao, 2021). Based on current studies of privacy protection, we expanded privacy protection issues among youths to the information system of digital travel platforms. We intended to explore the relationships between protection motivation, privacy concern, and privacy protection behavior among young online taxi users. Thus, we listed the following research questions:

Research Question 1: What is the status of protection motivation and privacy protection behavior among young people?
Research Question 2: Does protection motivation affect privacy concerns and privacy protection behavior among young people?
Research Question 3: What is the level of privacy knowledge of young users? Will it affect the relationship between privacy concerns and privacy protection behavior?

## Literature review

### Theories and hypotheses

Protection Motivation Theory (PMT) uses the social cognition perspective to examine an individual's behavior when faced with threats (Rogers, 1975). Following a series of research, PMT described its coping strategies in detail and categorized the motivation to self-protect from threats into two cognitive assessment processes: threat assessment (including perceived susceptibility and perceived severity) and coping assessment (including self-efficacy and response efficacy). Based on the assessment results of its cognitive threat, individuals may choose to engage in protection behavior (Rippetoe and Rogers, 1987). In terms of the PMT cognitive assessment processes, Witte argued that perceptual susceptibility and perceived severity described an individual's cognition of severity and possibility of a threatening occurrence, i.e., a perceived threat (Witte, 1992). Technology Threat Avoidance Theory (TTAT) further proposed that perceived threat was determined by predisposed variables of perceptual susceptibility and perceived severity. The perceived degree of potential threat initiated by technology would affect subjects' attitudes and behavior (Liang and Xue, 2009). This study combined PMT, TTAT, and other related research and intended to investigate the relationships between perceived threat, self-efficacy, response efficacy, privacy concern, and privacy protection behavior among youths who utilized DTP.

### Perceived threat, self-efficacy, response efficacy, and privacy concern

The predisposed variables of privacy concern, i.e., perceived threat, self-efficacy, and response efficacy, were used to measure the information privacy concerns. “Perceived threat” was defined as an individual's expected negative consequence of a certain technique, product, or even behavior, which affects the desire and motivation to take protective behavior (De Zwart et al., 2009). Therefore, this study used it to measure the perceived threat to personal information privacy among youths who utilized digital travel platforms. Self-efficacy is defined as an individual's capability to carry out expected behavior. Bandura asserted that self-efficacy was the perceived belief in individuals' capability to organize and execute the action process of established achievements (Bandura, 1977). Self-efficacy was the core concept of social psychology, which illustrated the belief in individuals' ability to execute behaviors successfully, and was critical to the explanation of subjective motivation. This study used self-efficacy as an attribute of youths' capability and confidence in protecting personal privacy from intrusion. Response efficacy was identified as the perceptual ability to reduce the risk effectively. The higher the belief that individuals benefit from protective behavior, the greater the motivation for engaging in such behavior (Maddux and Rogers, 1983), and an adaptive response to engaging in such protective behavior is capable of protecting themselves and others (Hanus and Wu, 2016).

The terminology and concept of privacy concern gradually appeared in academic fields due to the rapid development of information technology, which raised the issue of privacy protection and related research. Culnan argued that when an individual releases personal information to a certain organization, the issue of privacy concerns arises regarding how it will use and protect the information (Culnan, 1993). Information privacy concern refers to an inherent worry of information privacy loss, which was often applied to the research of predicting users' privacy protection behavior (Smith et al., 1996). Privacy concerns echoed the awareness of how service providers collect, restore, and use personal information obtained from customers (Sheng et al., 2008). Previous studies revealed that the worry about information privacy leakage significantly influenced the attitude and behavior of social media platforms (Adhikari and Panda, 2018). In addition, studies delineated that potential variables of protection motivation, such as perceived threat, self-efficacy, and response efficacy, tended to affect an individual's information privacy concern. Youn identified the perception of threat as a decisive factor in the internet privacy concern among youths (Youn, 2009). According to an empirical study of users' self-disclosure on the socialized internet, the greater the perceived risk, the higher the privacy concern (Chen, 2013).

Self-efficacy was another significant predisposing factor of privacy concern, which predicted the intention of taking protective behavior. Another study of accurate advertising push and consumers' privacy concerns found a positive correlation between self-efficacy in preventing privacy leakage from accurate advertising and privacy concern (Yu and Yang, 2019). Finally, a medical big data cloud study confirmed the significant positive relationship between self-efficacy and privacy concerns (Wu, 2020). Based on the above evidence, the following hypotheses were proposed:

H1. Self-efficacy has a positive influence on personal information privacy concerns.
H2. Response efficacy has a positive influence on personal information privacy concerns.
H3. A perceived threat has a positive influence on personal information privacy concerns.

### Privacy concern and privacy protection behavior

An empirical study of internet fraud confirmed that an increase in victims' predicted online privacy concerns tended to amplify privacy protection behavior (Chen et al., 2017). In addition, a related study of privacy protection delineated that users of socialized media tended to employ various modes of privacy protection behavior due to a high level of privacy concern (Feng and Xie, 2014). A similar Singapore study based on broadened planned behavior theory also found that the level of privacy doubt magnified the intention of online privacy protection (Ho et al., 2017). Another study on college students' privacy protection behavior verified that their privacy concerns about the WeChat APP influenced their privacy protection behavior significantly and positively (Xie and Karan, 2019). In order to examine the relationship between information privacy concerns and privacy protection behavior among youths, the following hypothesis was proposed:

H4. Privacy concern has a positive influence on privacy protection behavior.

### Indirect effect of privacy concern

In order to explore the predisposing factors of youths' privacy concerns, which affect privacy protection behavior among socialized internet users, a pragmatic study demonstrated that an indirect effect of privacy concern did exist in the relationships between perceived threat, self-efficacy, and privacy protection behavior (Hanus and Wu, 2016). Another study on the privacy protection behavior among Sina MicroBlog users also verified this indirect effect between perceived threat and privacy protection behavior; however, no indirect effect was found between the relationships of self-efficacy/response efficacy and privacy safety protection behavior (Wang et al., 2019). In addition, a Malaysian study of young socialized media users validated that perceived threat, self-efficacy, and response efficacy indirectly affect privacy protection behavior through privacy concerns (Adhikari and Panda, 2018). In order to verify the indirect effect of privacy concerns, the following hypotheses were proposed:

H5. Privacy concern mediates the relationship between perceived threats and privacy protection behavior.
H6. Privacy concern mediates the relationship between self-efficacy and privacy protection behavior.
H7. Privacy concern mediates the relationship between response efficacy and privacy protection behavior.

### Moderating effect of privacy knowledge

Privacy knowledge is a latent variable that could be flexibly elevated with refinement and training, thus reflecting its moderating characteristic. The results of a quasi-experimental study on the development of intelligent mobile phone APP software for privacy knowledge showed that APP users tended to pay more attention to their private personal information and use active protection means (Gerber et al., 2018). Similar research on children's digital literacy training revealed that the boost in training cost led to a decline in their personal information disclosure, which means children paid more attention to protecting their personal information privacy after training and tended to acquire protective actions (Desimpelaere et al., 2020). Knowledge regulated the relationship between privacy concerns and privacy protection behavior to a certain degree. How do young online taxi users comprehend the extent of privacy and information safety settings in the digital travel software they are using in their daily lives? Will it affect their protective manners? In order to verify these questions, the following hypotheses were proposed:

H8a. Privacy knowledge moderates the relationship between privacy concerns and privacy protection behavior.
H8b. Privacy knowledge groups moderate the relationship between privacy concerns and privacy protection behavior.

**Figure 1:**
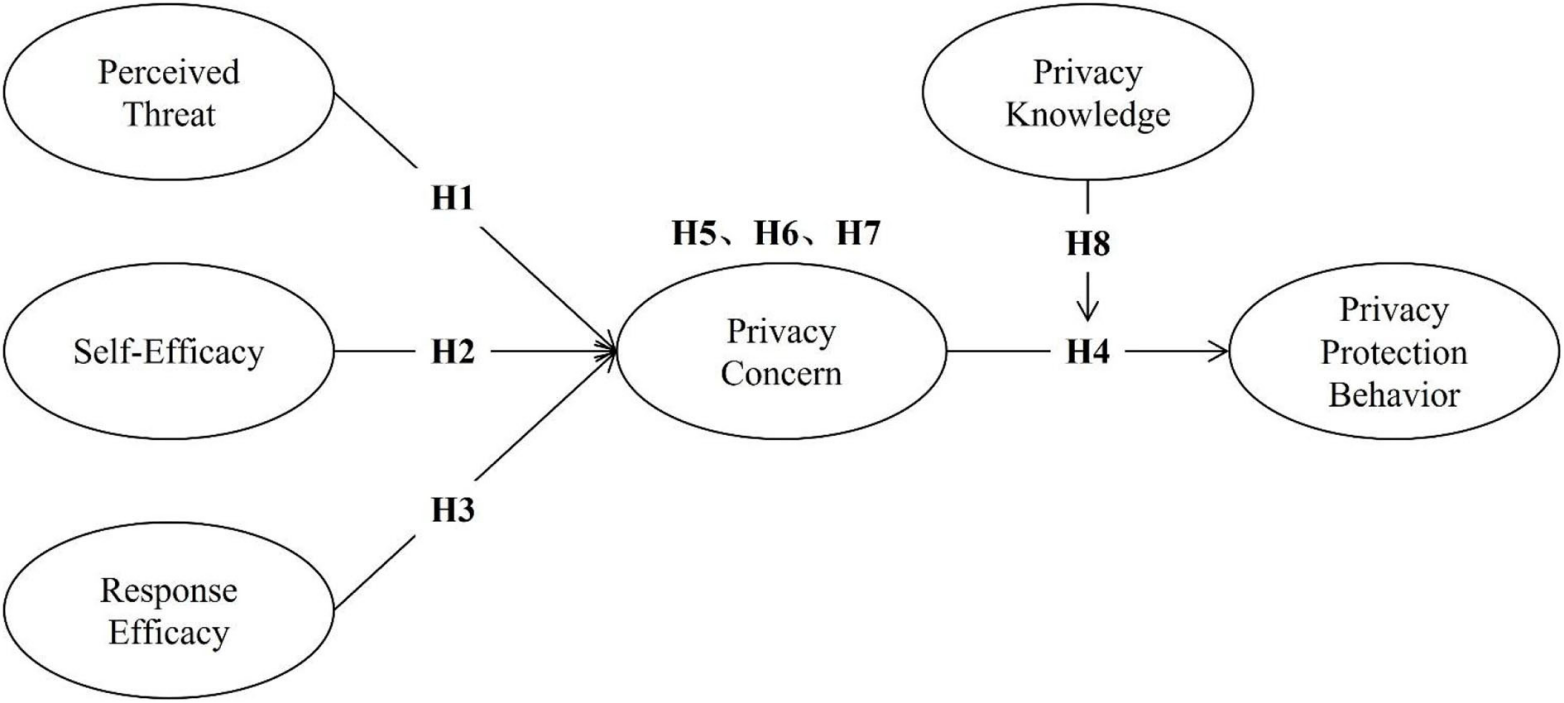
The framework of the research model.

Figure 1 summarizes the research model of the study.

## Research design

### Data collection and implementation

According to the regulation of *the “Medium and Long Term Youth Development Plan (2016-2035)”* released by the CPC Central Committee and the State Council, these study subjects were limited to Chinese youths aged 18 to 35 who employ DTP. Questionnaire Star was utilized to sketch the questionnaire and distribute it *via* WeChat Moments on August 17 and 30, 2021. A total of 507 subjects responded to the survey, excluding 66 invalid subjects and responses. A total of 441 subjects remained, with a sample qualification rate of 86.9%. This study adopted SPSS v23.0 for descriptive analyses, and AMOS v23.0 was used for confirmatory factor analyses and research hypotheses testing.

This study consisted of six dimensions, i.e., perceived threat, self-efficacy, response efficacy, privacy concern, privacy protection behavior, and privacy knowledge. Except for privacy, knowledge was segregated by dichotomized categories (yes, no, don't know), and a Likert 7-point scale was used for measuring the other variables (1 = totally disagree, 7 = totally agree). In order to ensure the reliability and validity of the questionnaire, a small-scale pilot test was conducted, and the tested subjects' opinions on questioning, sentencing, and wording were for modifications. In addition, several experts and scholars were invited for content validity checks and revision. The final version of the questionnaire consisted of six dimensions and 27 measurement indicators. The Appendix A shows the detailed questionnaire measurement items. The structure of the survey is shown in Table 1.

**Table 1:** Research variables and sources.

**Variables**	**Sources**	**No. of items**
Perceived threat	(Johnston and Warkentin, 2010; Qi and Li, 2018)	3
Self-efficacy	(Schwarzer et al., 1999; Youn, 2009)	5
Response efficacy	(Workman et al., 2008)	3
Privacy concern	(Taylor et al., 2009; Adhikari and Panda, 2018)	4
Privacy protection behavior	(Hanus and Wu, 2016)	4
Privacy knowledge	(Park and Jang, 2014; Masur et al., 2017; Rosenthal et al., 2020)	8

## Statistical analysis and hypothesis test

### Descriptive analysis

Female respondents accounted for 61.2 vs. 38.8% of males. Regarding age allocation, respondents aged 18–25 accounted for 46.7%, 26–30 34.9%, and 31–35 18.4%. The majority of respondents were students (39.9%), enterprise employees (35.1%), personnel of public institutions, and other occupations accounted for 25%. Education level of an undergraduate degree accounted for the majority of 49%. Regarding monthly income, 69.8% reported less than 8,000 RMB, and 30.2% over 8,000 RMB.

SEM-AMOS was used for the confirmatory factor analysis of the research model. All standardized factor loadings (STD) were greater than 0.6, Cronbach's α and composite reliability (CR) were higher than 0.7, and the convergence effect (AVE) was higher than 0.5, which illustrated the excellent reliability and validity of the research model. In addition, Table 2 identified all the AVE square roots as being greater than the correlation coefficients between the variables, which indicated outstanding discriminant validity among the variables. The Appendix B shows the detailed measurement model reliability.

**Table 2:** Reliability, convergent and discriminant validities of the research model.

**Variable**	**FL**	**CR**	**AVE**	**PT**	**SEEF**	**REEF**	**PC**	**PPB**
PT	0.733~0.857	0.694	0.639	**0.799**				
SEEF	0.765~0.880	0.700	0.694	0.254	**0.833**			
REEF	0.775~0.881	0.639	0.700	0.268	0.745	**0.837**		
PC	0.824~0.887	0.705	0.705	0.603	0.366	0.379	**0.840**	
PPB	0.627~0.796	0.525	0.525	0.428	0.563	0.499	0.560	**0.725**

FL, factor loadings; CR, composite reliability; AVE, average variance extracted. SEEF, self-efficacy; REEF, response efficacy; PT, perceived threat; PC, privacy concern; PPB, privacy protection behavior.

### Structural model

Based on the calculations of AMOS, related indices of model fitness were as follows: Normed Chi-square (χ^2^/DF) = 2.947, GFI = 0.902, NFI = 0.922, IFI = 0.947, TLI (NNFI) = 0.937, CFI = 0.947, RMSEA = 0.067. All the indexes were in a reasonable range, which confirmed that the fitness of the research model was acceptable.

### Path analysis and hypothesis test

**Figure 2:**
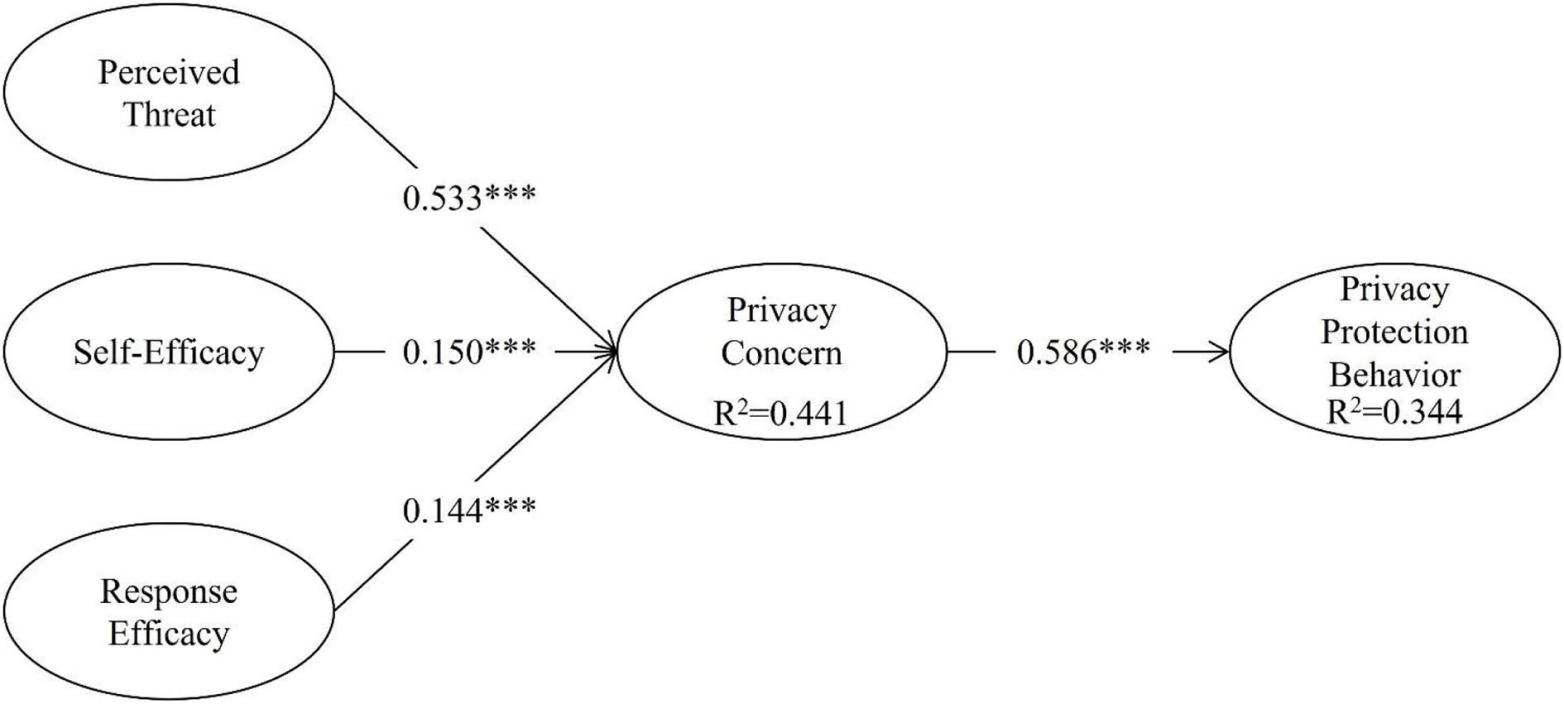
Path coefficients of the structural equation model. *p < 0.05; **p < 0.01, ***p < 0.001.

Figure 2 illustrates the regression coefficients as follows: perceived threat (*β* = 0.533, *p* < 0.001), self-efficacy (*β* = 0.144, *p* < 0.05), and response efficacy (*β* = 0.150, *p* < 0.05), which significantly affect privacy concern (R^2^=0.441). In addition, privacy concern (*β* = 0.586, p < 0.001) significantly affects privacy protection behavior (R^2^ = 0.344). Therefore, hypotheses 1 4 were accepted to various degrees.

### Indirect effect of privacy concern

Bootstrapping 5,000 times was utilized to check the indirect effect, with the bias-corrected 95% CI and percentile 95% CI not including 0. Table 3 delineated the significant total effect and total indirect effect of perceived threat, self-efficacy, and response efficacy on privacy protection behavior (*p* < 0.05), which confirmed the indirect effect of privacy concern. Thus, hypothesis 5/6/7 were supported.

**Table 3:** Indirect effects of privacy concerns.

**Hypothesis**	**Effect**	**P. E**.	**C.P**.			**Bias-corrected 95% CI**		**Percentile 95% CI**		**Result**
			**SE**	**Z**	***P***	**LL**	**UL**	**LL**	**UL**	
H5	Total effect SEEF → PPB	0.200	0.042	4.762	0.000	0.127	0.291	0.121	0.285	Accept support
	TIE SEEF → PPB	0.200	0.042	4.762	0.000	0.127	0.291	0.121	0.285
H6	Total effect REEF → PPB	0.223	0.054	4.130	0.000	0.131	0.342	0.125	0.337	Accept support
	TIE REEF → PPB	0.223	0.054	4.130	0.000	0.131	0.342	0.125	0.337
H7	Total effect PT → PPB	0.452	0.073	6.192	0.000	0.311	0.600	0.311	0.600	Accept support
	TIE PT → PPB	0.452	0.073	6.192	0.000	0.311	0.600	0.311	0.600	

### Moderating effect of privacy knowledge

The moderating effect of privacy knowledge was one of the key interpretations of this study. The following specific procedures followed:

Step 1: The scores of eight items were summed up for a total score. All the total scores were divided into three groups: high (top 27 percentile), medium, and low (bottom 27 percentile) scores, based on Cureton (1957) proposal. In order to maintain statistical power, the difference only between high- and low-score groups (120 subjects each) was calculated. The independent *t-*test identified significant differences between high- and low-privacy knowledge level groups (t = −30.933).

Step 2: Grouping regression and identity tests were conducted using AMOS software. In order to examine the significant difference, the combination of high- and low-score groups was designated as the constraint model (all the parameters are equal) and compared with the default model (without any restriction) (Wen et al., 2012). Table 4 delineated significant results of grouping regression: the Chi-square value change of the constraint model ($\chi_{95\text{\%},1\text{df}}^{2}$ = 8.941 > 3.84) with *p* = *0.003*, which concluded that significant privacy knowledge moderates the relationship between privacy concern and privacy protection behavior. In order to consolidate the credibility of the findings, the following outcomes were identified:

The *p* values of both models were less than 0.001, and CMIN/*df* values were < 3.
The baseline comparison found significant differences in the NFI, RFI, IFI, TLI, and CFI values.
RMSEA indexes of the models were unequal (0.059 vs. 0.061).

**Table 4:** Grouping regression of the constraint model vs. the default model.

**Model**	**NPAR**	**CMIN**	***df***	***P***	**CMIN/** ***df***
Default	51	144.159	57	<0.001	2.529
Constraint	50	153.100	58	<0.001	2.640
	**NFI**	**RFI**	**IFI**	**TLI**	**CFI**
Default	0.926	0.891	0.954	0.931	0.953
Constraint	0.921	0.886	0.950	0.926	0.949
	**RMSEA**	**Lo 90**	**Hi 90**	**PCLOSE**
Default	0.059	0.047	0.071	0.102
Constraint	0.061	0.049	0.073	0.057

Thus, the default and constraint models were not matched, i.e., hypothesis 8a should be accepted.

Step 3: Data from grouping regression demonstrated greater mean values of privacy concern and privacy protection behavior in the high privacy knowledge group than that of the low privacy knowledge group, with regression coefficients of 0.371 (high privacy knowledge group) vs. 0.620 (low privacy knowledge group), which means the impact of the moderating effect among the high privacy knowledge group was significantly lower than that of their counterparts (data not shown in Table 4). Therefore, hypothesis 8b should be rejected.

## Conclusion and discussion

### Conclusion

This study expanded privacy protection theory and context to digital travel platforms that youths employ in their daily lives, work, and social contact. Based on a comprehensive of understanding the privacy protection behavior of contemporary youth online taxi users, this study offered coping strategies from subjective and objective dimensions of youths' privacy protection and hoped digital society could protect the personal information and privacy of youths. The conclusions are as follows:

A perceived threat, self-efficacy, and response efficacy positively affected privacy concerns;
Privacy concerns positively affected privacy protection behavior. Youths tended to have a higher level of privacy concern (with a mean value of 5.187 over 7) and used countermeasures to protect their privacy, such as fake names and shutting off location services;
Privacy concern was an intermediate factor in the relationships between perceived threat, self-efficacy, response efficacy, and privacy protection behavior;
Privacy knowledge moderates the relationship between privacy concerns and privacy protection behavior. The mean values of privacy concern and privacy protection behavior in the high privacy knowledge group were significantly greater than those of their counterparts. However, the predictive power of privacy concern on privacy protection behavior in the high privacy knowledge group was significantly less than that of their counterparts.

### Discussion

Perceived threat, self-efficacy, and response efficacy were significant variables in predicting the relationship between privacy concerns and privacy protection behavior among youths utilizing DTP. Of which, the perceived threat was identified as the main predictive factor of privacy concern, followed by response efficacy and self-efficacy. In addition, the mean values of these variables were greater than their average scores, which denoted that youth online taxi users did not trust digital travel platforms. The implications of this finding are 2 fold: on the one hand, at the level of the impact of perceived risk on privacy concern, the results of this study echo previous studies on Internet use and the privacy concern of social media use among youths (Youn, 2009; Ho et al., 2017). Although youths of internet aborigines handled digital travel platforms in their daily lives constantly, they still sharply noticed the threat of digital technology to personal information, data, and privacy.

On the other hand, previous studies have suggested that self-efficacy is unrelated to privacy concerns (Yao et al., 2007). Contrary to previous studies, the statistical results of the two kinds of efficacy reported in our study indicate that self-efficacy and response efficacy have significant effects on privacy concerns. It is precisely because the youths are technologically proficient and thus believe that they are able to effectively protect their private information. These findings exposed self-confidence in information technology among contemporary youths, i.e., they are capable of employing cutting-edge technological gadgets to protect their privacy.

Are youths concerned about their privacy? Youths are the most active and vital force in society. In the era of privacy transparency, the entire society is questioning privacy concerns among youths. It is valuable and meaningful to examine whether youths pay attention to the information privacy of DTP or not. This study found an average score of privacy concern of 5.187 out of 7, which revealed a high level of privacy concern about digital travel platforms among the youth of online taxi users. It is noteworthy that privacy concerns not only directly influenced the privacy protection behavior of the youths but also functioned as an indirect factor between the relationships of perceived threat, self-efficacy, response efficacy, and privacy protection behavior. This finding is in line with the study of Lee et al. (2017). They suggest that privacy concerns have a positive impact on online privacy protection behavior among young people, which means that privacy concerns are an important element of privacy management for youth that cannot be ignored.

One of the imperative findings of this study was that the predictive power of privacy concern on privacy protection behavior among the high privacy knowledge group was significantly less than that of the low privacy knowledge group. Schwarzer et al. (1999) suggest that self-efficacy pertains to optimistic beliefs about coping with a large variety of stressors. However, excessive optimism can lead individuals to develop “optimism bias.” Weinstein asserted that individuals tended to believe in having a greater opportunity to encounter active events than inactive ones, and negative experience with privacy protection might depress an individual's enthusiasm for acquiring protective action (Weinstein, 1980), which explained the logic of this finding. Sharot (2012) demonstrated the existence of optimism bias in human society through an experimental study and argued that optimism bias is a result of the evolution of the human brain, which can subconsciously change the subject's behavior and enhance individual wellbeing, but optimism bias may also cause blind optimism due to a lack of crisis awareness and reduce the individual's sense of prevention. Xu (2011) confirmed the optimism bias of social network users. People usually believe they may be less vulnerable to privacy risks than others.

Similarly, another study also shows that users generally believe that negative events such as privacy leaks or information trafficking are less likely to happen to them (Campbell et al., 2007). In line with the above studies, our study also found the existence of so-called “optimistic bias” among the high privacy knowledge group. Due to the phenomenon of optimistic bias, individuals with high privacy knowledge tend to assume that they cannot confront threats more often than their counterparts. Therefore, they had a high level of privacy concern but a low level of privacy protection behavior. On the contrary, individuals with low privacy knowledge tended to lack IT awareness and skill, thus paying less attention to privacy and protective settings. Because they were unfamiliar with the degree of threat and its damage, which led to anxiety, they tended to enhance privacy concerns and adopt an aggressive protection mode when facing threats. This finding supports earlier research on the optimism bias of privacy risk (Kim and Hancock, 2015; Metzger and Suh, 2017). In addition, the results illustrated an inadequate understanding and familiarity with the privacy settings of digital travel platforms among youths, and approximately two-thirds were college students, meaning the knowledge of privacy settings was irrelevant to education level. The probable rationale was that youths tended to operate DTP when they needed online taxi-hailing but neglected the concern of privacy settings in their daily lives.

Youths should enhance their coping abilities with privacy risks. Firstly, intensifying the threat perception could effectively promote their concern for personal information and encourage them to adopt positive protective action on DTP. Secondly, individuals with extraordinary self-efficacy tended to adopt more active protective measures when applying digital travel platforms—for example, downloading travel software *via* an authorized APP store instead of a homepage link and avoiding clicking offensive websites to prevent possible intrusion of personal information. In addition, youths are able to promote response efficacy by paying more attention to related information about upholding privacy protection, awakening the coping ability of risky behavior, conducting adaptive training, such as conscious training on specific cases (i.e., party role-playing), and exercising prompt response aptitude. Finally, youths must recognize that enhancing their level of privacy knowledge is the most important method of preventing privacy threats. The fortification of skills and knowledge on privacy risk can improve privacy protection behavior and reduce the probability of infringement. Youths should improve their identification of various privacy risks and realize how to avoid them (Marcolin et al., 2000). Paying attention to the various elements of information safety, obtaining safety education and related training, enriching the knowledge of personal privacy protection, and keeping risk awareness of preparing for a rainy day are the required courses for youths to elevate personal information literacy.

From the perspective of platform self-discipline, a digital travel platform (an immediate information processor) is responsible for protecting users' information safety, particularly youths' information privacy. DTP must visibly declare the critical content of its privacy protection policy straightforwardly and clearly illustrate what kind of personal information was collected and how it was used. Thus, platform users are able to know fairly well how to raise awareness of privacy management. In addition, explicit, informed consent is the core principle of personal privacy protection and a basic maxim to comply with. Digital travel platforms should carefully respect youths' informational self-determination, exercise withdrawal, and obtain users' re-authorization as they employ the platform.

From the perspective of industry supervision, relevant government authorities should establish proprietary specifications for digital travel platform information privacy protection as soon as possible. Due to economies of scale and capital-seduced self-discipline failure, digital travel platforms tend to exhibit opportunistic motivations of so-called “management malfeasance.” From the perspective of the legal guarantee, the legislations of the Civil Code, Personal Information Protection Law, and Data Security Law protected Chinese citizens' rights and interests in various information privacy matters effectively. When serious threats occur, youths should actively exercise their legal rights to defend personal information and privacy.

Nowadays, instead of sticking to a specific subject, communication research should focus on all walks of life (Schiller, 2018). Privacy is a multifaceted social problem, and youths are the backbone of society. Therefore, research on youths' consumption of DTP and privacy protection behaviors tended to have more academic value and space. This study explored the impact factors of digital travel platform utilization and privacy protection behavior among youths from a quantitative perspective. Further studies can examine the concerns and attitudes toward the privacy protection of DTP among youths. In addition, this study tried to examine youths' modes of acquiring knowledge of personal information privacy protection using test questions but failed to report the actual knowledge level objectively. Therefore, further studies are needed to measure multiple dimensions of knowledge regarding personal information protection.

## Data availability statement

The original contributions presented in the study are included in the article/Supplementary material, further inquiries can be directed to the corresponding author.

## Author contributions

XM: guidance of research design, statistical analyses, and proof writing. BF: research designer and executor, conducting statistical analyses, and first draft writing. All authors contributed to the article and approved the submitted version.

## Funding

This study was supported by Chongqing Academy of Social Sciences (Grant No. 2022NDYB112).

## Conflict of interest

The authors declare that the research was conducted in the absence of any commercial or financial relationships that could be construed as a potential conflict of interest.

## Publisher's note

All claims expressed in this article are solely those of the authors and do not necessarily represent those of their affiliated organizations, or those of the publisher, the editors and the reviewers. Any product that may be evaluated in this article, or claim that may be made by its manufacturer, is not guaranteed or endorsed by the publisher.
