# Supplementary material for: Online taxi users' optimistic bias: China youths' digital travel and information privacy protection
Source: Front Psychol. 2022 Nov 22;13:1049925. doi: 10.3389/fpsyg.2022.1049925 (PMC9723153; doi:10.3389/fpsyg.2022.1049925)
Supplement: Supplementary file 1 [file Table_1.docx]

**Appendix A**

Questionnaire Measurement Items.

| Variables | Items |
| --- | --- |
| Perceived Threat | I think that DTP has the risk of revealing privacy information  I think that privacy information on DTP is vulnerable to illegal use  I think that the leakage of private information on DTP is very serious |
|  |  |
| Self-Efficacy | I am confident that I could protect the privacy information in DTP  I am confident that I could stop DTP from violating my privacy information  I am confident that I could use the security settings of DTP to protect my privacy information  I am confident that I could control the collection and use of my privacy information by DTP  I am confident that I could stop DTP from tracking and leaking my privacy information |
|  |  |
| Response Efficacy | Efforts to keep my privacy information safe are effective  The security settings function to protect my privacy information safe are effective  The privacy policy to protect my privacy information safe are effective |
| Privacy Concern | I am concerned that my privacy information in DTP could be misused  I am concerned that my privacy information in DTP could be lost（like location, phone number）  I am concerned that my privacy information in DTP could be stolen while being transferred（like address, account）  I am concerned that my privacy information in DTP could be used in a manner I am unaware of（like recording video） |
|  |  |
| Privacy Protect Behavior | I would deliberately misrepresent privacy information in DTP  I would regularly clear the cache in DTP（like location history）  I would set up privacy number protection in DTP  I would disable the real-time location feature of DTP |
| Privacy Knowledge | **Knowledge of information collection and processing：**  DTP only collect personal information that is needed to deliver the service.  DTP’s "trip recording function" is only enabled when the passenger authorises it  DTP delete personal data after a pre-defined period  When DTP has a privacy policy, it means DTP will not share your information with other companies  Turn off the "personalized advertising recommendation function" in DTP and you will no longer receive advertising marketing messages  Using a pseudonym or anonymity on DTP makes yourself more difficult to identify  Knowledge of location：  When you use DTP, it is legal to collect your location and privacy information  When you deactivate GPS on your phone, your location cannot be tracked by DTP |
|  |  |
|  |  |

DTP, Digital travel platform.

**Appendix B**

Measurement model reliability.

| Constructs | Measurement item | Standardized factor loadings | Cronbachs α | CR | AVE |
| --- | --- | --- | --- | --- | --- |
| Perceived Threat | PT1 | 0.803 | 0.836 | 0.841 | 0.639 |
|  | PT2 | 0.857 |  |  |  |
|  | PT3 | 0.733 |  |  |  |
| Self-Efficacy | SEEF1 | 0.811 | 0.919 | 0.919 | 0.694 |
|  | SEEF2 | 0.861 |  |  |  |
|  | SEEF3 | 0.765 |  |  |  |
|  | SEEF4 | 0.843 |  |  |  |
|  | SEEF5 | 0.88 |  |  |  |
| Response Efficacy | REEF1 | 0.775 | 0.872 | 0.875 | 0.7 |
|  | REEF2 | 0.85 |  |  |  |
|  | REEF3 | 0.881 |  |  |  |
| Privacy Concern | PC1 | 0.824 | 0.904 | 0.905 | 0.705 |
|  | PC2 | 0.887 |  |  |  |
|  | PC3 | 0.852 |  |  |  |
|  | PC4 | 0.792 |  |  |  |
| Privacy Protection Behavior | PPB1 | 0.627 | 0.808 | 0.814 | 0.525 |
|  | PPB2 | 0.796 |  |  |  |
|  | PPB3 | 0.708 |  |  |  |
|  | PPB4 | 0.755 |  |  |  |

CR, composite reliability; AVE, average variance extracted.

SEEF, self-efficacy; REEF, response efficacy; PT, perceived threat; PC, privacy concern; PPB, privacy protection behavior.
